# Poly[[diaqua­(μ-4,4′-bipyridine *N*,*N*′-di­oxide-κ^2^ *O*:*O*′)(μ-terephthalato-κ^2^ *O* ^1^:*O* ^4^)cobalt(II)] 4,4′-bipyridine *N*,*N*′-dioxide monosolvate]

**DOI:** 10.1107/S1600536812040056

**Published:** 2012-09-26

**Authors:** Xin Ge, Shu-Yan Song

**Affiliations:** a State Key Laboratory of Rare Earth Resource Utilization, Changchun Institute of Applied Chemistry, Chinese Academy of Sciences, Changchun 130022, People’s Republic of China

## Abstract

In the title compound, {[Co(C_8_H_4_O_4_)(C_10_H_8_N_2_O_2_)(H_2_O)_2_]·C_10_H_8_N_2_O_2_}_*n*_, the Co^II^ atom, lying on an inversion center, is hexa­coordinated in a distorted octa­hedral geometry defined by two O atoms from two terephthalate (tp) ligands, two O atoms from two 4,4′-bipyridine *N*,*N*′-dioxide (bpydo) ligands and two water mol­ecules. The coordinated tp and bpydo ligands and uncoordinated bpydo mol­ecule all have an inversion center. The Co^II^ atoms are connected by the tp and bpydo ligands into a layer parallel to (111). In the crystal, O—H⋯O hydrogen bonds link the uncoordinated bpydo mol­ecules and the layers into a three-dimensional supra­molecular structure. Intra­layer O—H⋯O hydrogen bonds and π–π inter­actions [centroid-to-centroid distances = 3.6643 (13) and 3.8048 (13) Å] are also observed.

## Related literature

For the design of supra­molecular structures containing metal ions and organic ligands, see: Liao *et al.* (2008 ▶); Wang *et al.* (2008 ▶). For a related structure, see: Su *et al.* (2009 ▶).
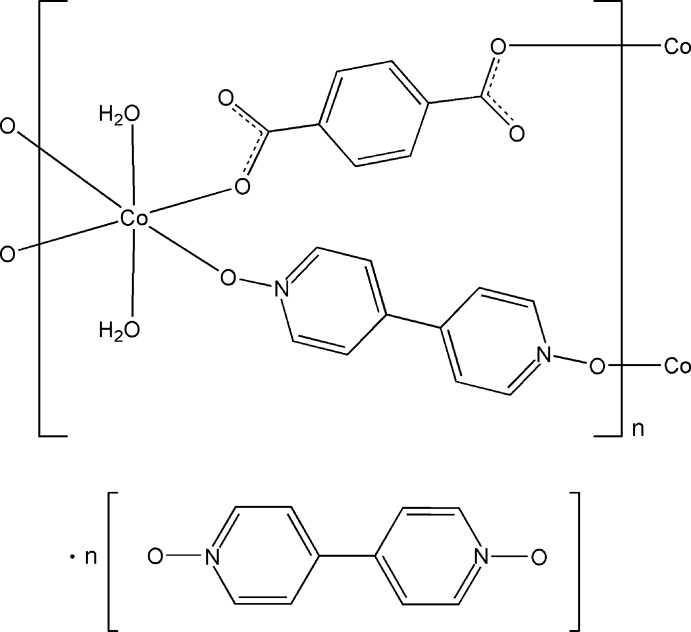


## Experimental

### 

#### Crystal data

[Co(C_8_H_4_O_4_)(C_10_H_8_N_2_O_2_)(H_2_O)_2_]·C_10_H_8_N_2_O_2_
*M* *_r_* = 635.44
Triclinic, 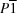

*a* = 7.3883 (10) Å
*b* = 9.1788 (13) Å
*c* = 9.8054 (13) Å
α = 81.312 (2)°
β = 82.200 (2)°
γ = 79.301 (2)°
*V* = 641.92 (15) Å^3^
*Z* = 1
Mo *K*α radiation
μ = 0.74 mm^−1^
*T* = 293 K
0.27 × 0.24 × 0.20 mm

#### Data collection

Bruker APEXII CCD diffractometer
Absorption correction: multi-scan (*SADABS*; Bruker, 2001 ▶) *T*_min_ = 0.825, *T*_max_ = 0.866
3574 measured reflections
2516 independent reflections
2340 reflections with *I* > 2σ(*I*)
*R*_int_ = 0.017

#### Refinement

*R*[*F* ^2^ > 2σ(*F* ^2^)] = 0.031
*wR*(*F* ^2^) = 0.081
*S* = 1.05
2516 reflections
202 parameters
2 restraints
H atoms treated by a mixture of independent and constrained refinement
Δρ_max_ = 0.48 e Å^−3^
Δρ_min_ = −0.33 e Å^−3^

### 

Data collection: *APEX2* (Bruker, 2007 ▶); cell refinement: *SAINT* (Bruker, 2007 ▶); data reduction: *SAINT*; program(s) used to solve structure: *SHELXTL* (Sheldrick, 2008 ▶); program(s) used to refine structure: *SHELXTL*; molecular graphics: *XP* in *SHELXTL*; software used to prepare material for publication: *SHELXTL*.

## Supplementary Material

Crystal structure: contains datablock(s) global, I. DOI: 10.1107/S1600536812040056/hy2589sup1.cif

Structure factors: contains datablock(s) I. DOI: 10.1107/S1600536812040056/hy2589Isup2.hkl

Additional supplementary materials:  crystallographic information; 3D view; checkCIF report

## Figures and Tables

**Table 1:** Hydrogen-bond geometry (Å, °)

*D*—H⋯*A*	*D*—H	H⋯*A*	*D*⋯*A*	*D*—H⋯*A*
O5—H5*A*⋯O2	0.84 (2)	1.92 (2)	2.755 (2)	170 (2)
O5—H5*B*⋯O3^i^	0.85 (2)	1.85 (2)	2.660 (2)	158 (3)

Symmetry code: (i) 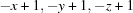.

**Fig. 1.**
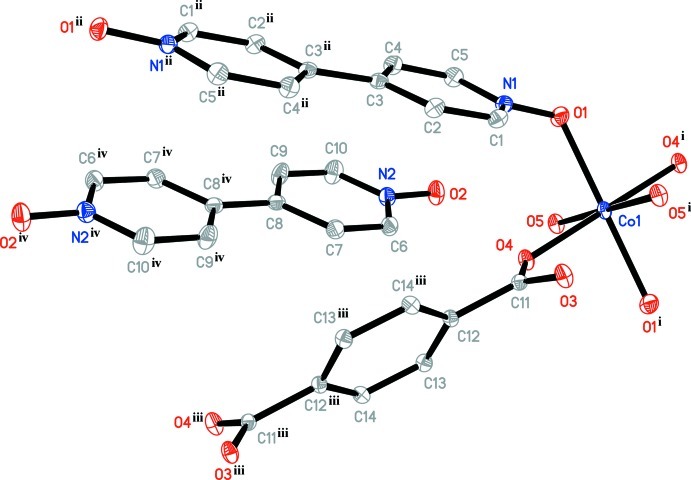
The asymmetric unit of the title compound. Displacement ellipsoids are drawn at the 30% probability level. H atoms have been omitted for clarity. [Symmetry codes: (i) 1 - x, 1 - y, 1 - z; (ii) 1 - x, 2 - y, -z; (iii) 2 - x, 1 - y, -z; (iv) 2 - x, 2 - y, -z.]

**Fig. 2.**
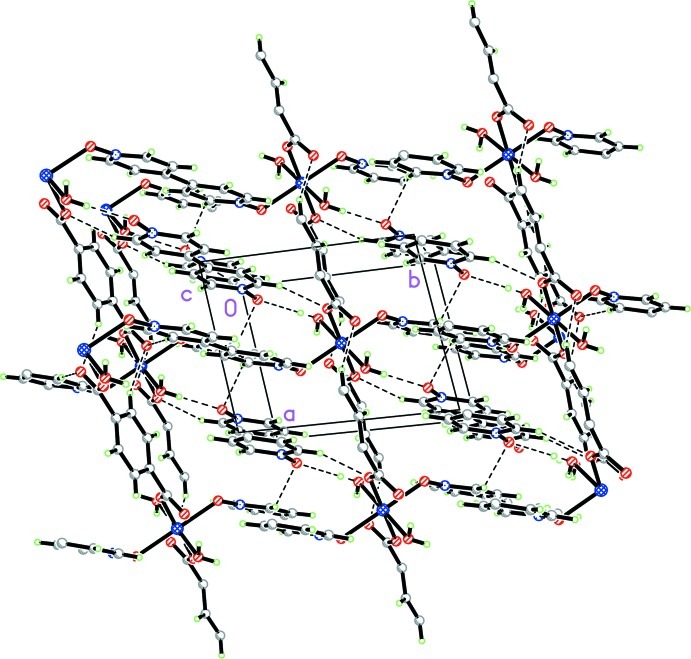
View of the three-dimensional structure of the title compound, built by hydrogen bonds (dashed lines).

## supplementary crystallographic information

### Comment

Much progress has been achieved in the design of supramolecular structures containing metal–organic molecules during recent years (Liao *et al.*, 2008; Wang *et al.*, 2008). Multifunctional ligands can link metal ions into one-, two- or three-dimensional structures, and in this context, aromatic carboxylates and 4,4'-bipyridine *N*,*N*'-dioxide have been used successfully to synthesize such materials.

As shown in Fig. 1, the coordination environment of the Co^II^ atom, lying on an inversion center, can be described as distorted octahedral, defined by four O atoms in the equatorial plane from two terephthalate (tp) ligands and two 4,4'-bipyridine *N*,*N*'-dioxide (bpydo) ligands, and two water molecules in the axial positions. The bond distances and angles are normal (Su *et al.*, 2009). The Co^II^ atoms are connected by the tp and bpydo ligands, forming a layer structure parallel to (111). O—H···O hydrogen bonds (Table 1) link the uncoordinated bpydo molecules and the layers into a three-dimensional supramolecular structure (Fig. 2). Intralayer O—H···O hydrogen bonds and π–π interactions [centroid–centroid distances = 3.6643 (13) and 3.8048 (13) Å] are also observed.

### Experimental

A mixture of terephthalic acid (0.1 mmol, 0.017 g), 4,4'-bipyridine *N*,*N*'-dioxide (0.2 mmol, 0.038 g), cobalt nitrate (0.1 mmol, 0.030 g), H_2_O (5 ml) and dimethylformamide (15 ml) was stirred at 358 K for 10 min. The mixture was filtrated and pink block crystals of the title compound were isolated after evaporation of the solvent.

### Refinement

H atoms on C atoms were positioned geometrically and refined as riding atoms, with C—H = 0.93 Å and with *U*_iso_(H) = 1.2*U*_eq_(C). H atoms of water molecule were located in a difference Fourier map and refined with a restraint of O—H = 0.85 (1) Å and with *U*_iso_(H) = 1.5*U*_eq_(O).

### Crystal data

**Table:**

[Co(C_8_H_4_O_4_)(C_10_H_8_N_2_O_2_)(H_2_O)_2_]·C_10_H_8_N_2_O_2_	*Z* = 1
*M**_r_* = 635.44	*F*(000) = 327
Triclinic, *P*1	*D*_x_ = 1.644 Mg m^−^^3^
Hall symbol: -P 1	Mo *K*α radiation, λ = 0.71073 Å
*a* = 7.3883 (10) Å	Cell parameters from 2899 reflections
*b* = 9.1788 (13) Å	θ = 2.3–26.1°
*c* = 9.8054 (13) Å	µ = 0.74 mm^−^^1^
α = 81.312 (2)°	*T* = 293 K
β = 82.200 (2)°	Block, pink
γ = 79.301 (2)°	0.27 × 0.24 × 0.20 mm
*V* = 641.92 (15) Å^3^	

### Data collection

**Table:**

Bruker APEXII CCD diffractometer	2516 independent reflections
Radiation source: fine-focus sealed tube	2340 reflections with *I* > 2σ(*I*)
Graphite monochromator	*R*_int_ = 0.017
φ and ω scans	θ_max_ = 26.1°, θ_min_ = 2.1°
Absorption correction: multi-scan (*SADABS*; Bruker, 2001)	*h* = −8→9
*T*_min_ = 0.825, *T*_max_ = 0.866	*k* = −10→11
3574 measured reflections	*l* = −8→12

### Refinement

**Table:**

Refinement on *F*^2^	Primary atom site location: structure-invariant direct methods
Least-squares matrix: full	Secondary atom site location: difference Fourier map
*R*[*F*^2^ > 2σ(*F*^2^)] = 0.031	Hydrogen site location: inferred from neighbouring sites
*wR*(*F*^2^) = 0.081	H atoms treated by a mixture of independent and constrained refinement
*S* = 1.05	*w* = 1/[σ^2^(*F*_o_^2^) + (0.0389*P*)^2^ + 0.3924*P*] where *P* = (*F*_o_^2^ + 2*F*_c_^2^)/3
2516 reflections	(Δ/σ)_max_ < 0.001
202 parameters	Δρ_max_ = 0.48 e Å^−^^3^
2 restraints	Δρ_min_ = −0.33 e Å^−^^3^

### Special details

**Table:**

Geometry. All esds (except the esd in the dihedral angle between two l.s. planes) are estimated using the full covariance matrix. The cell esds are taken into account individually in the estimation of esds in distances, angles and torsion angles; correlations between esds in cell parameters are only used when they are defined by crystal symmetry. An approximate (isotropic) treatment of cell esds is used for estimating esds involving l.s. planes.
Refinement. Refinement of F^2^ against ALL reflections. The weighted R-factor wR and goodness of fit S are based on F^2^, conventional R-factors R are based on F, with F set to zero for negative F^2^. The threshold expression of F^2^ > 2sigma(F^2^) is used only for calculating R-factors(gt) etc. and is not relevant to the choice of reflections for refinement. R-factors based on F^2^ are statistically about twice as large as those based on F, and R- factors based on ALL data will be even larger.

### Fractional atomic coordinates and isotropic or equivalent isotropic displacement parameters (Å^2^)

**Table:**

	*x*	*y*	*z*	*U*_iso_*/*U*_eq_	
Co1	0.5000	0.5000	0.5000	0.01630 (11)	
C1	0.3852 (3)	0.7380 (2)	0.2083 (2)	0.0221 (4)	
H1	0.3528	0.6439	0.2142	0.027*	
C2	0.4275 (3)	0.8156 (2)	0.08038 (19)	0.0209 (4)	
H2	0.4249	0.7727	0.0007	0.025*	
C3	0.4745 (2)	0.9580 (2)	0.06822 (18)	0.0186 (4)	
C4	0.4684 (3)	1.0181 (2)	0.1918 (2)	0.0233 (4)	
H4	0.4925	1.1145	0.1887	0.028*	
C5	0.4271 (3)	0.9365 (2)	0.3176 (2)	0.0242 (4)	
H5	0.4246	0.9781	0.3988	0.029*	
C6	0.8804 (3)	0.7935 (2)	0.2615 (2)	0.0270 (4)	
H6	0.8534	0.6980	0.2909	0.032*	
C7	0.9285 (3)	0.8359 (2)	0.1234 (2)	0.0264 (4)	
H7	0.9313	0.7689	0.0606	0.032*	
C8	0.9735 (3)	0.9770 (2)	0.0743 (2)	0.0217 (4)	
C9	0.9655 (3)	1.0700 (2)	0.1762 (2)	0.0317 (5)	
H9	0.9958	1.1649	0.1496	0.038*	
C10	0.9150 (3)	1.0265 (2)	0.3131 (2)	0.0344 (5)	
H10	0.9102	1.0920	0.3777	0.041*	
C11	0.6942 (2)	0.44694 (19)	0.21107 (18)	0.0164 (4)	
C12	0.8539 (2)	0.47312 (19)	0.10187 (18)	0.0162 (3)	
C13	1.0328 (2)	0.46045 (19)	0.13839 (18)	0.0175 (4)	
H13	1.0549	0.4345	0.2308	0.021*	
C14	1.1778 (2)	0.4865 (2)	0.03689 (18)	0.0179 (4)	
H14	1.2970	0.4770	0.0617	0.021*	
N1	0.3901 (2)	0.79675 (17)	0.32562 (16)	0.0206 (3)	
N2	0.8716 (2)	0.88841 (18)	0.35576 (18)	0.0254 (4)	
O1	0.3567 (2)	0.71908 (15)	0.44822 (14)	0.0266 (3)	
O2	0.8186 (2)	0.84874 (17)	0.48725 (15)	0.0350 (4)	
O3	0.57776 (18)	0.37620 (15)	0.18300 (14)	0.0232 (3)	
O4	0.68909 (17)	0.50355 (14)	0.32145 (12)	0.0194 (3)	
O5	0.67652 (19)	0.59404 (15)	0.59885 (14)	0.0225 (3)	
H5A	0.719 (3)	0.6689 (19)	0.555 (2)	0.034*	
H5B	0.605 (3)	0.626 (3)	0.6676 (17)	0.034*	

### Atomic displacement parameters (Å^2^)

**Table:**

	*U*^11^	*U*^22^	*U*^33^	*U*^12^	*U*^13^	*U*^23^
Co1	0.01820 (19)	0.02037 (19)	0.01144 (18)	−0.00652 (13)	0.00000 (13)	−0.00296 (13)
C1	0.0249 (9)	0.0193 (9)	0.0237 (10)	−0.0039 (7)	−0.0069 (8)	−0.0034 (7)
C2	0.0255 (9)	0.0216 (9)	0.0175 (9)	−0.0037 (7)	−0.0071 (7)	−0.0046 (7)
C3	0.0171 (8)	0.0206 (9)	0.0179 (9)	−0.0003 (7)	−0.0048 (7)	−0.0026 (7)
C4	0.0280 (10)	0.0213 (9)	0.0218 (10)	−0.0048 (7)	−0.0024 (8)	−0.0057 (7)
C5	0.0301 (10)	0.0250 (10)	0.0188 (9)	−0.0041 (8)	−0.0018 (8)	−0.0081 (8)
C6	0.0300 (10)	0.0204 (9)	0.0310 (11)	−0.0082 (8)	0.0042 (8)	−0.0062 (8)
C7	0.0288 (10)	0.0210 (9)	0.0302 (11)	−0.0063 (8)	0.0028 (8)	−0.0086 (8)
C8	0.0179 (9)	0.0185 (9)	0.0289 (11)	−0.0020 (7)	−0.0020 (7)	−0.0048 (7)
C9	0.0451 (13)	0.0205 (10)	0.0318 (12)	−0.0132 (9)	−0.0024 (10)	−0.0026 (8)
C10	0.0504 (14)	0.0267 (11)	0.0304 (12)	−0.0155 (10)	−0.0029 (10)	−0.0080 (9)
C11	0.0182 (8)	0.0164 (8)	0.0137 (8)	−0.0020 (6)	−0.0012 (7)	−0.0008 (7)
C12	0.0190 (8)	0.0162 (8)	0.0141 (9)	−0.0044 (6)	0.0011 (7)	−0.0055 (7)
C13	0.0218 (9)	0.0200 (8)	0.0113 (8)	−0.0042 (7)	−0.0021 (7)	−0.0028 (7)
C14	0.0166 (8)	0.0217 (9)	0.0167 (9)	−0.0039 (7)	−0.0026 (7)	−0.0051 (7)
N1	0.0212 (8)	0.0229 (8)	0.0159 (8)	−0.0011 (6)	−0.0012 (6)	−0.0009 (6)
N2	0.0265 (8)	0.0251 (8)	0.0252 (9)	−0.0074 (7)	0.0007 (7)	−0.0043 (7)
O1	0.0343 (8)	0.0249 (7)	0.0168 (7)	−0.0022 (6)	0.0022 (6)	0.0016 (5)
O2	0.0473 (9)	0.0337 (8)	0.0253 (8)	−0.0170 (7)	0.0073 (7)	−0.0058 (6)
O3	0.0241 (7)	0.0301 (7)	0.0195 (7)	−0.0133 (6)	0.0022 (5)	−0.0095 (5)
O4	0.0221 (6)	0.0259 (7)	0.0123 (6)	−0.0093 (5)	0.0018 (5)	−0.0055 (5)
O5	0.0234 (7)	0.0282 (7)	0.0181 (7)	−0.0109 (6)	0.0008 (5)	−0.0048 (6)

### Geometric parameters (Å, º)

**Table:**

Co1—O4^i^	2.0865 (12)	C7—H7	0.9300
Co1—O4	2.0865 (12)	C8—C9	1.398 (3)
Co1—O5	2.0975 (13)	C8—C8^iii^	1.475 (4)
Co1—O5^i^	2.0976 (13)	C9—C10	1.364 (3)
Co1—O1^i^	2.1141 (13)	C9—H9	0.9300
Co1—O1	2.1141 (13)	C10—N2	1.356 (3)
C1—N1	1.350 (2)	C10—H10	0.9300
C1—C2	1.373 (3)	C11—O3	1.250 (2)
C1—H1	0.9300	C11—O4	1.263 (2)
C2—C3	1.397 (3)	C11—C12	1.510 (2)
C2—H2	0.9300	C12—C14^iv^	1.395 (2)
C3—C4	1.398 (3)	C12—C13	1.396 (2)
C3—C3^ii^	1.479 (4)	C13—C14	1.389 (3)
C4—C5	1.371 (3)	C13—H13	0.9300
C4—H4	0.9300	C14—C12^iv^	1.395 (2)
C5—N1	1.349 (3)	C14—H14	0.9300
C5—H5	0.9300	N1—O1	1.319 (2)
C6—N2	1.350 (3)	N2—O2	1.312 (2)
C6—C7	1.368 (3)	O5—H5A	0.84 (2)
C6—H6	0.9300	O5—H5B	0.85 (2)
C7—C8	1.395 (3)		
			
O4^i^—Co1—O4	180.0	C6—C7—H7	119.1
O4^i^—Co1—O5	90.46 (5)	C8—C7—H7	119.1
O4—Co1—O5	89.54 (5)	C7—C8—C9	115.04 (18)
O4^i^—Co1—O5^i^	89.54 (5)	C7—C8—C8^iii^	122.1 (2)
O4—Co1—O5^i^	90.46 (5)	C9—C8—C8^iii^	122.9 (2)
O5—Co1—O5^i^	180.00 (6)	C10—C9—C8	122.32 (19)
O4^i^—Co1—O1^i^	95.05 (5)	C10—C9—H9	118.8
O4—Co1—O1^i^	84.95 (5)	C8—C9—H9	118.8
O5—Co1—O1^i^	92.31 (6)	N2—C10—C9	120.4 (2)
O5^i^—Co1—O1^i^	87.69 (6)	N2—C10—H10	119.8
O4^i^—Co1—O1	84.95 (5)	C9—C10—H10	119.8
O4—Co1—O1	95.05 (5)	O3—C11—O4	126.25 (16)
O5—Co1—O1	87.69 (6)	O3—C11—C12	117.81 (15)
O5^i^—Co1—O1	92.31 (6)	O4—C11—C12	115.90 (15)
O1^i^—Co1—O1	180.0	C14^iv^—C12—C13	119.39 (16)
N1—C1—C2	120.77 (17)	C14^iv^—C12—C11	119.82 (15)
N1—C1—H1	119.6	C13—C12—C11	120.77 (15)
C2—C1—H1	119.6	C14—C13—C12	120.09 (16)
C1—C2—C3	120.92 (17)	C14—C13—H13	120.0
C1—C2—H2	119.5	C12—C13—H13	120.0
C3—C2—H2	119.5	C13—C14—C12^iv^	120.52 (16)
C2—C3—C4	116.56 (17)	C13—C14—H14	119.7
C2—C3—C3^ii^	121.9 (2)	C12^iv^—C14—H14	119.7
C4—C3—C3^ii^	121.5 (2)	O1—N1—C5	119.74 (16)
C5—C4—C3	120.68 (18)	O1—N1—C1	120.43 (16)
C5—C4—H4	119.7	C5—N1—C1	119.82 (16)
C3—C4—H4	119.7	O2—N2—C6	120.49 (16)
N1—C5—C4	121.11 (17)	O2—N2—C10	120.07 (17)
N1—C5—H5	119.4	C6—N2—C10	119.44 (18)
C4—C5—H5	119.4	N1—O1—Co1	121.82 (11)
N2—C6—C7	120.93 (18)	C11—O4—Co1	130.60 (11)
N2—C6—H6	119.5	Co1—O5—H5A	117.3 (17)
C7—C6—H6	119.5	Co1—O5—H5B	102.4 (17)
C6—C7—C8	121.82 (19)	H5A—O5—H5B	106 (2)
			
N1—C1—C2—C3	−0.8 (3)	C4—C5—N1—C1	−3.0 (3)
C1—C2—C3—C4	−2.5 (3)	C2—C1—N1—O1	−177.21 (16)
C1—C2—C3—C3^ii^	178.3 (2)	C2—C1—N1—C5	3.6 (3)
C2—C3—C4—C5	3.1 (3)	C7—C6—N2—O2	177.42 (19)
C3^ii^—C3—C4—C5	−177.7 (2)	C7—C6—N2—C10	−1.4 (3)
C3—C4—C5—N1	−0.5 (3)	C9—C10—N2—O2	−178.3 (2)
N2—C6—C7—C8	1.1 (3)	C9—C10—N2—C6	0.5 (3)
C6—C7—C8—C9	0.0 (3)	C5—N1—O1—Co1	−128.29 (15)
C6—C7—C8—C8^iii^	179.6 (2)	C1—N1—O1—Co1	52.5 (2)
C7—C8—C9—C10	−0.9 (3)	O4^i^—Co1—O1—N1	−171.81 (14)
C8^iii^—C8—C9—C10	179.5 (2)	O4—Co1—O1—N1	8.19 (14)
C8—C9—C10—N2	0.6 (4)	O5—Co1—O1—N1	97.52 (14)
O3—C11—C12—C14^iv^	42.0 (2)	O5^i^—Co1—O1—N1	−82.48 (14)
O4—C11—C12—C14^iv^	−135.93 (17)	O3—C11—O4—Co1	2.1 (3)
O3—C11—C12—C13	−139.35 (17)	C12—C11—O4—Co1	179.77 (11)
O4—C11—C12—C13	42.7 (2)	O5—Co1—O4—C11	170.87 (15)
C14^iv^—C12—C13—C14	−0.6 (3)	O5^i^—Co1—O4—C11	−9.13 (15)
C11—C12—C13—C14	−179.28 (16)	O1^i^—Co1—O4—C11	78.51 (15)
C12—C13—C14—C12^iv^	0.6 (3)	O1—Co1—O4—C11	−101.49 (15)
C4—C5—N1—O1	177.84 (17)		

Symmetry codes: (i) −*x*+1, −*y*+1, −*z*+1; (ii) −*x*+1, −*y*+2, −*z*; (iii) −*x*+2, −*y*+2, −*z*; (iv) −*x*+2, −*y*+1, −*z*.

### Hydrogen-bond geometry (Å, º)

**Table:**

*D*—H···*A*	*D*—H	H···*A*	*D*···*A*	*D*—H···*A*
O5—H5*A*···O2	0.84 (2)	1.92 (2)	2.755 (2)	170 (2)
O5—H5*B*···O3^i^	0.85 (2)	1.85 (2)	2.660 (2)	158 (3)

Symmetry code: (i) −*x*+1, −*y*+1, −*z*+1.

## References

[bb1] Bruker (2001). *SADABS* Bruker AXS Inc., Madison, Wisconsin, USA.

[bb2] Bruker (2007). *APEX2* and *SAINT* Bruker AXS Inc., Madison, Wisconsin, USA.

[bb3] Liao, C. Y., Chan, K. T., Chiu, P. L., Chen, C. Y. & Lee, H. M. (2008). *Inorg. Chim. Acta*, **361**, 2973–2978.

[bb4] Sheldrick, G. M. (2008). *Acta Cryst.* A**64**, 112–122. 10.1107/S0108767307043930 18156677

[bb5] Su, S.-Q., Guo, Z.-Y., Li, G.-H., Deng, R.-P., Song, S.-Y., Qin, C., Pan, C.-L., Guo, H.-D., Cao, F., Wang, S. & Zhang, H.-J. (2009). *Dalton Trans.* **39**, 9123–9130. 10.1039/c001655a 20820468

[bb6] Wang, G.-H., Li, Z.-G., Jia, H.-Q., Hu, N.-H. & Xu, J.-W. (2008). *Cryst. Growth Des.* **8**, 1932–1939.

